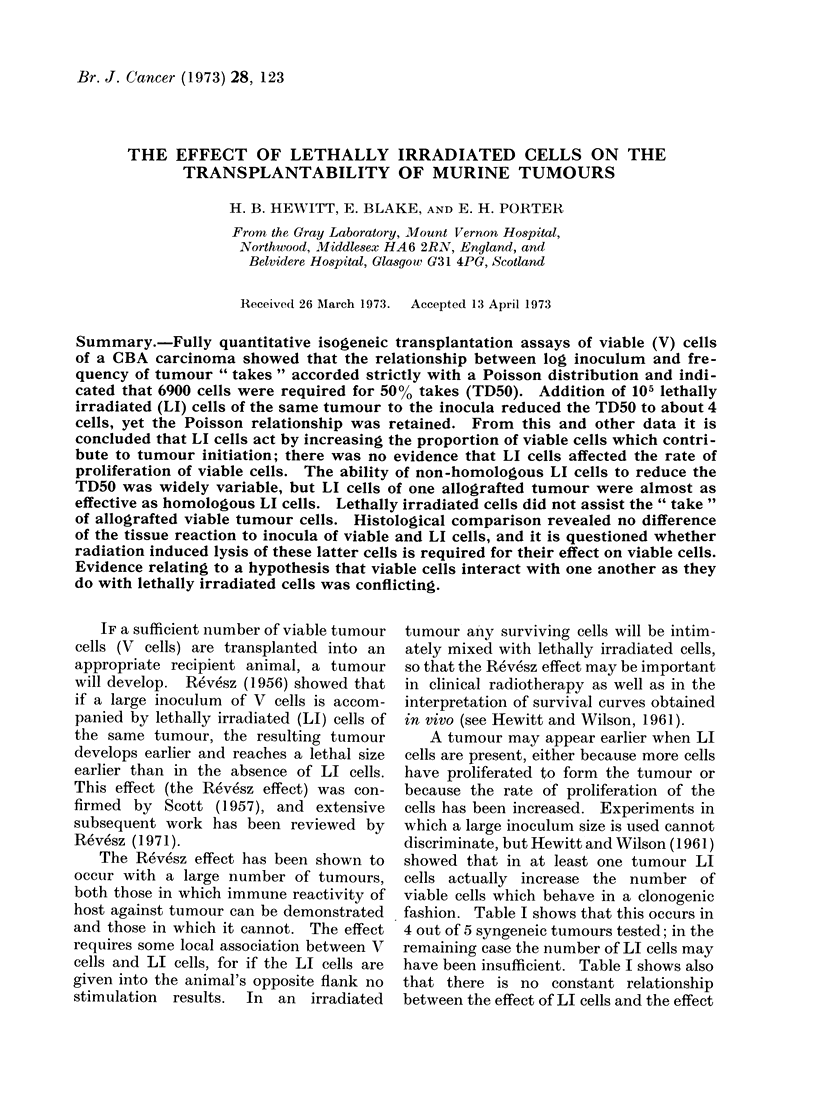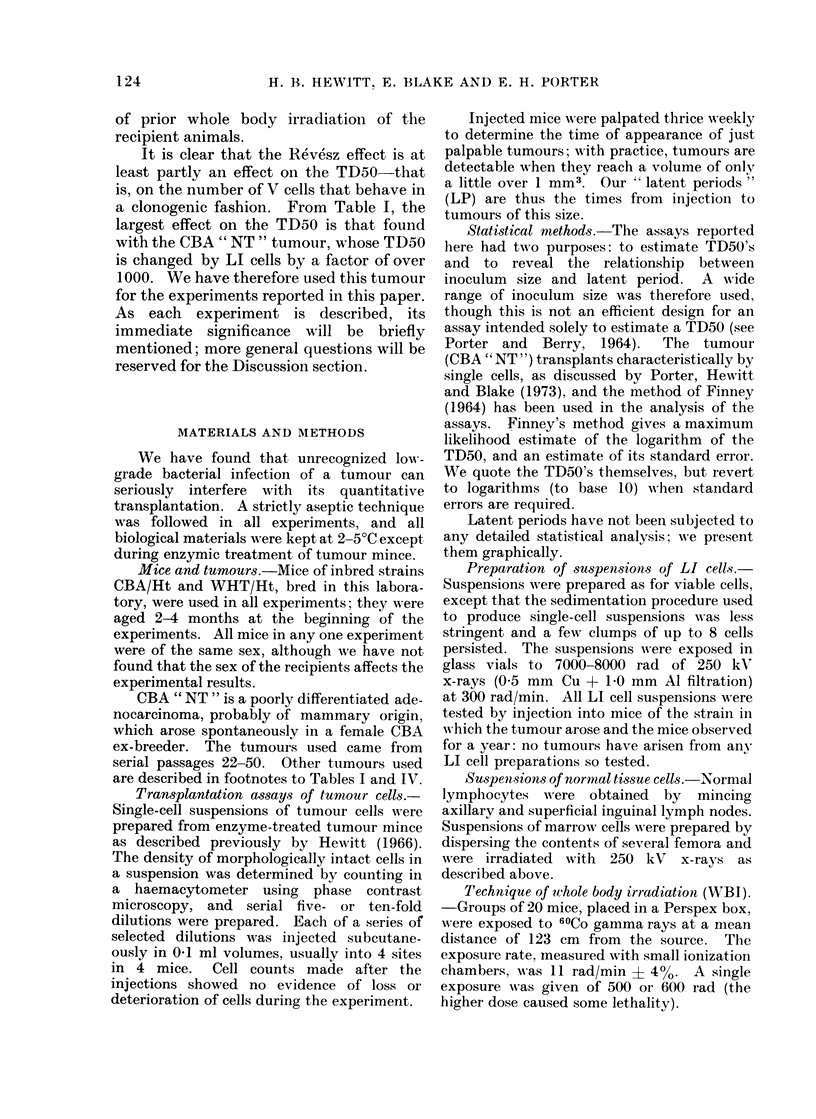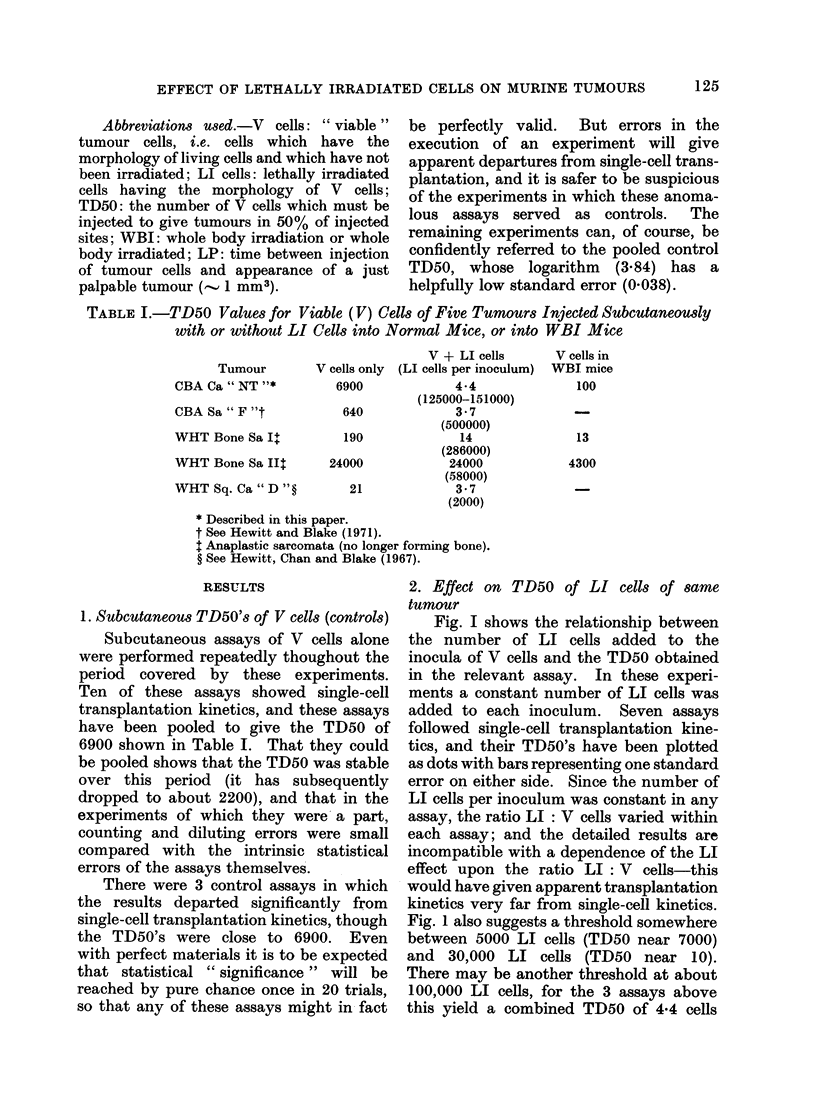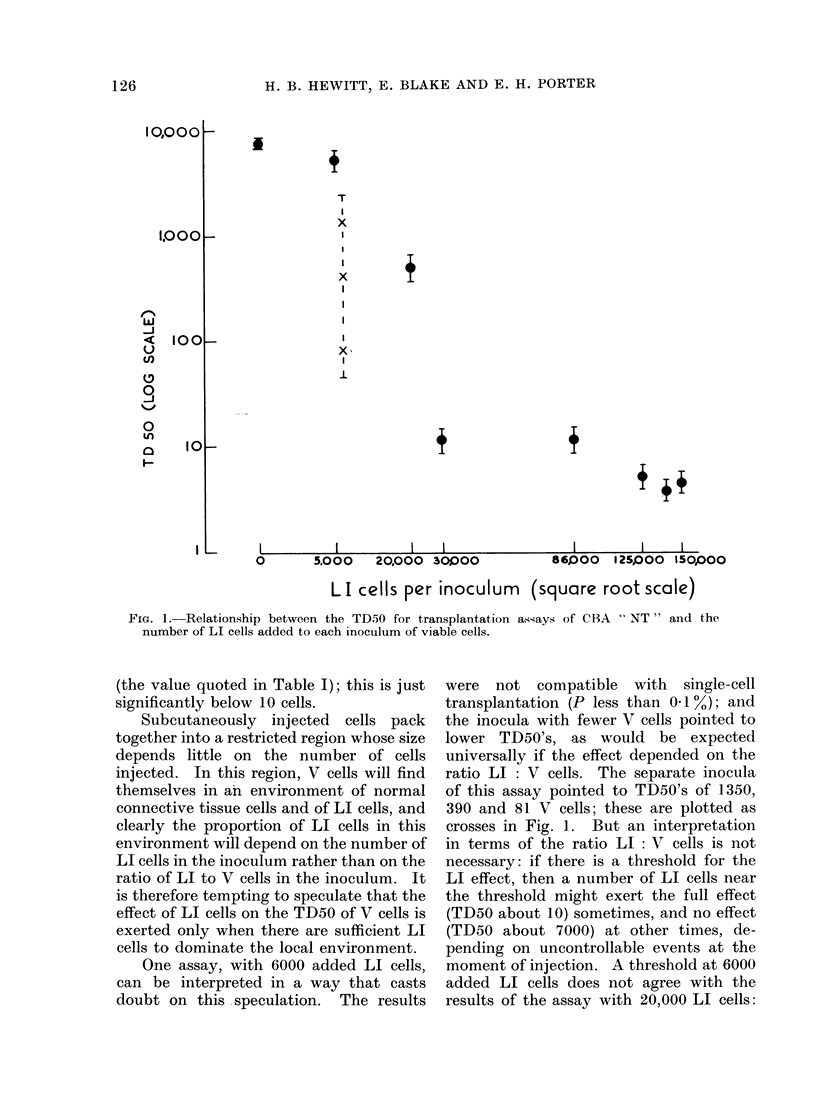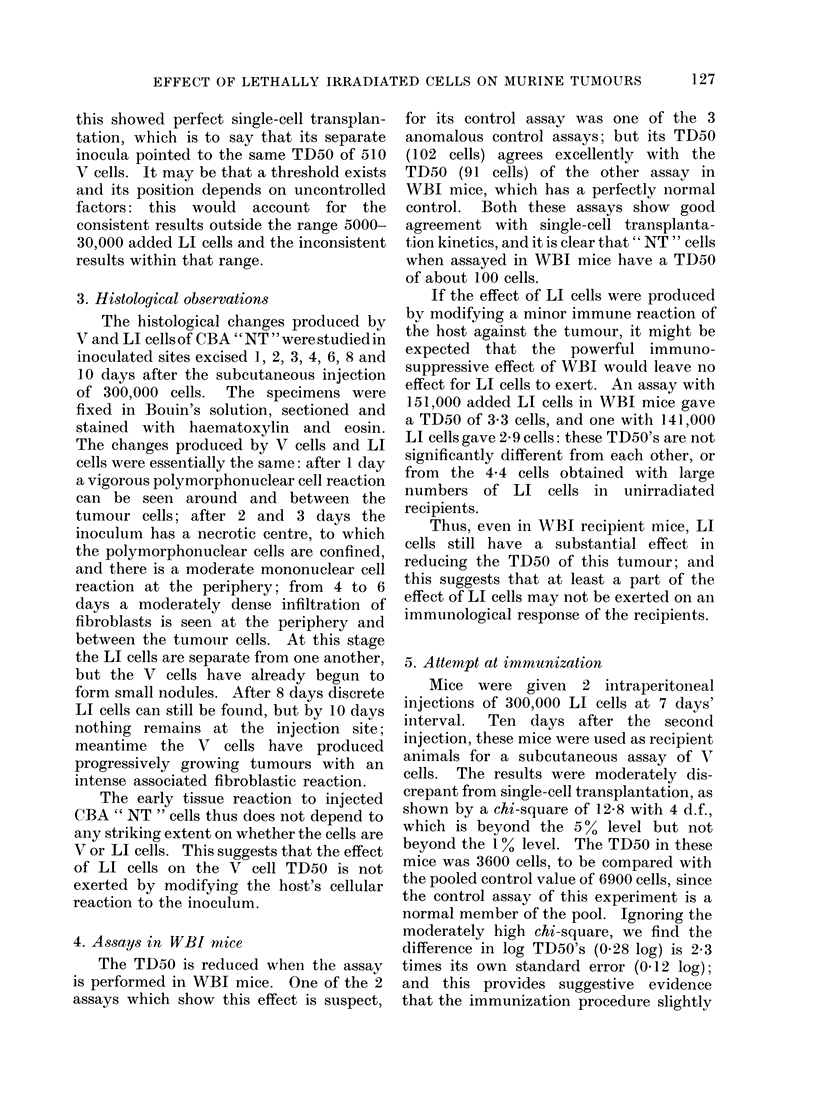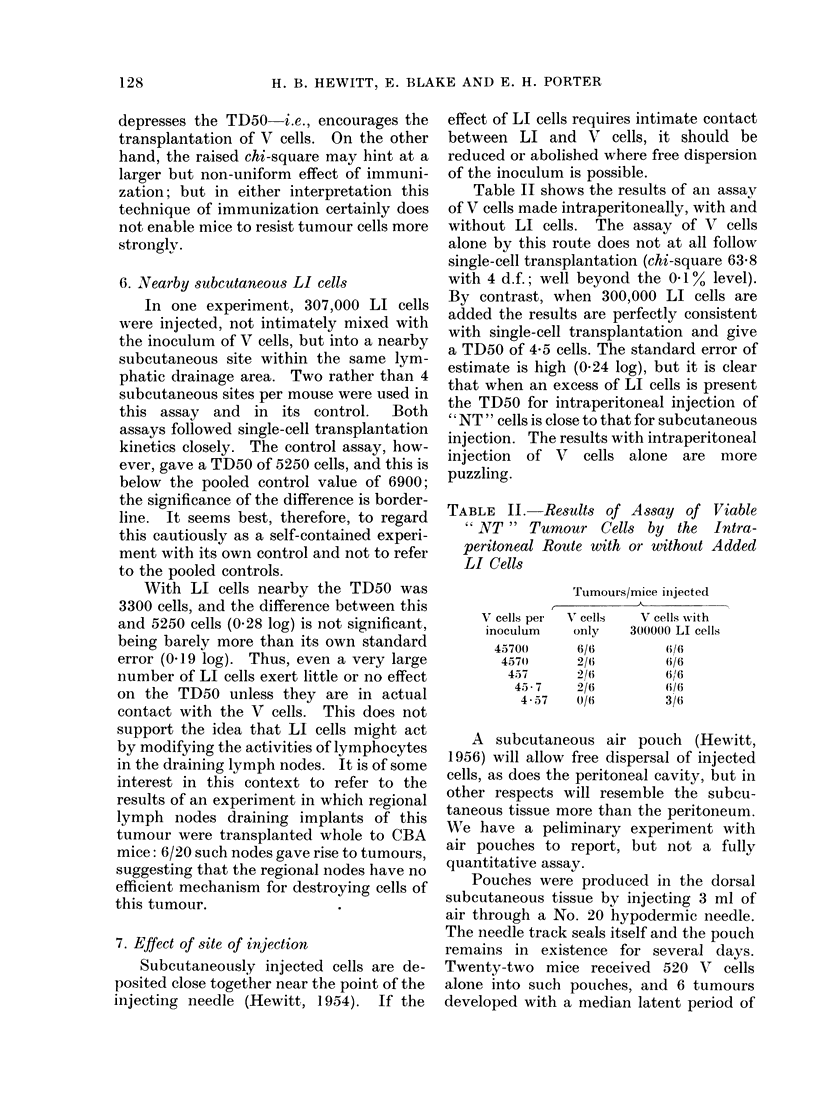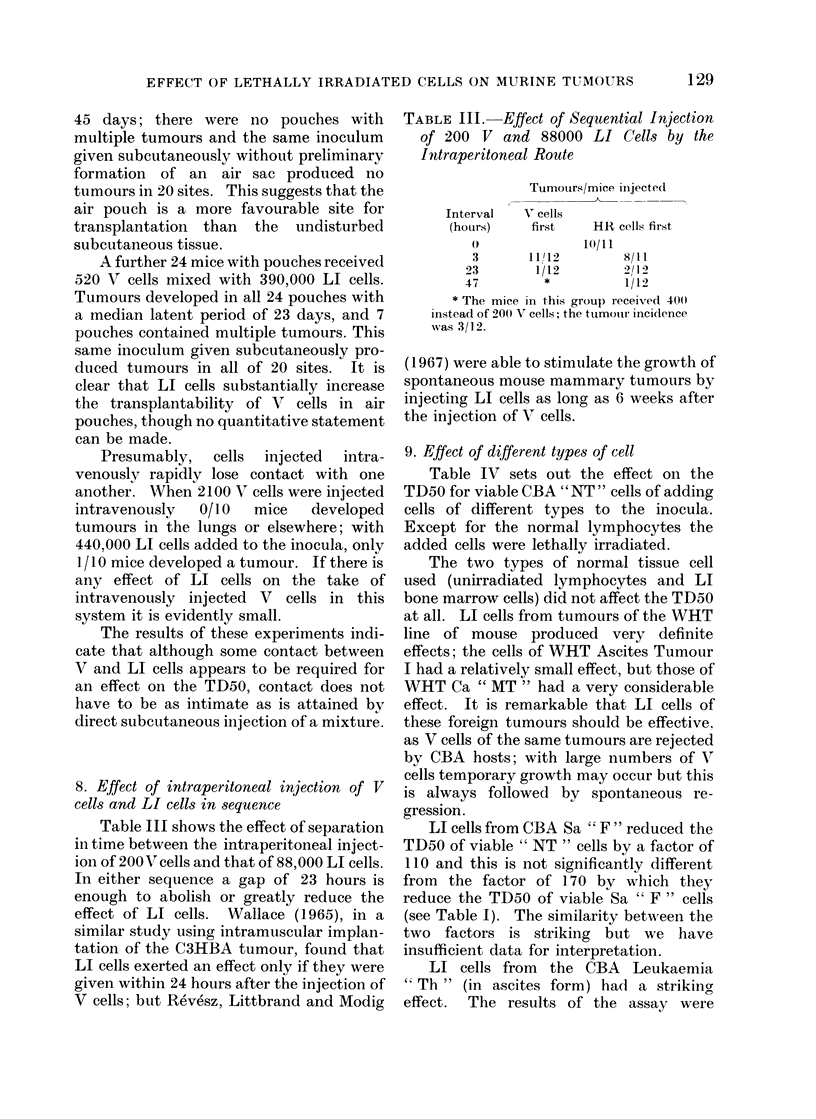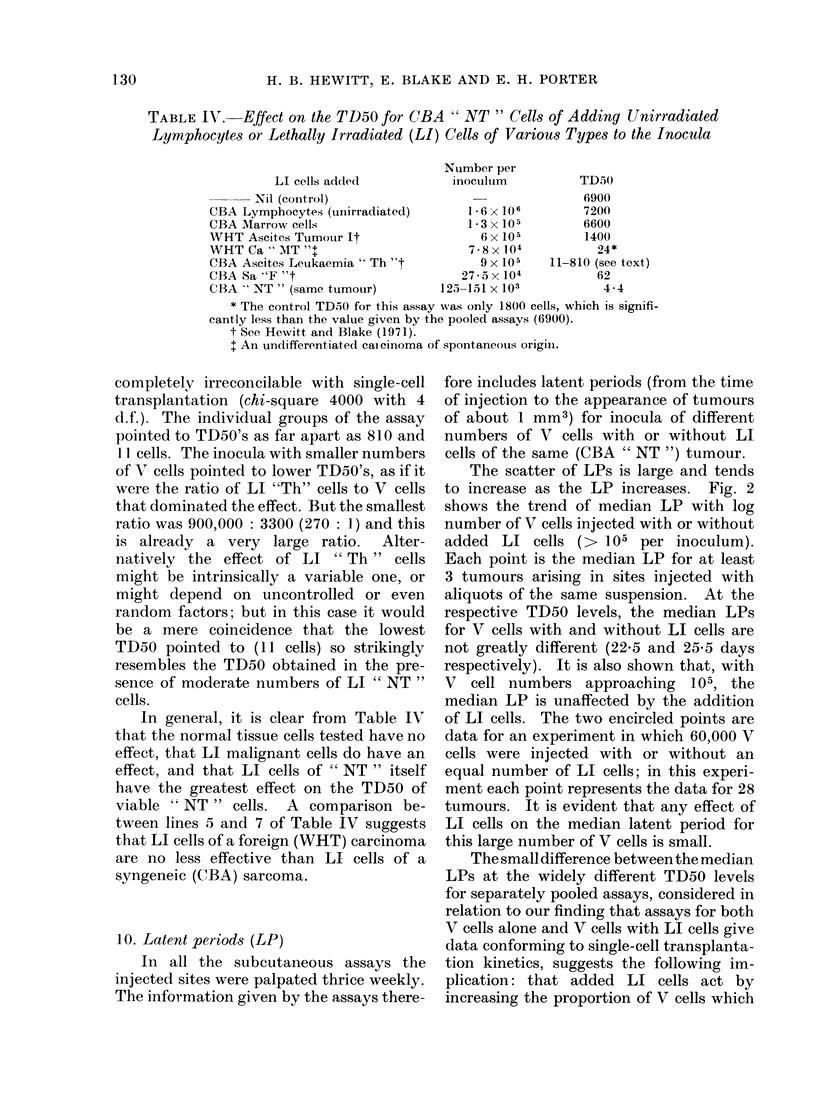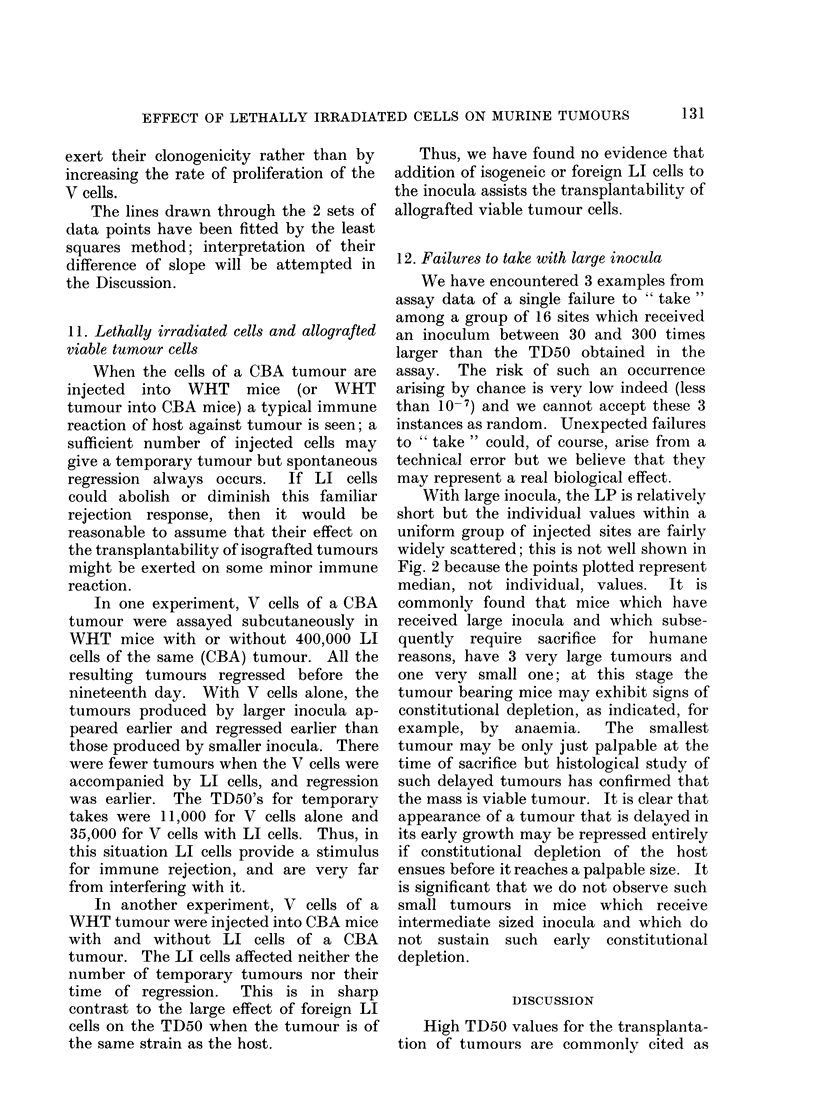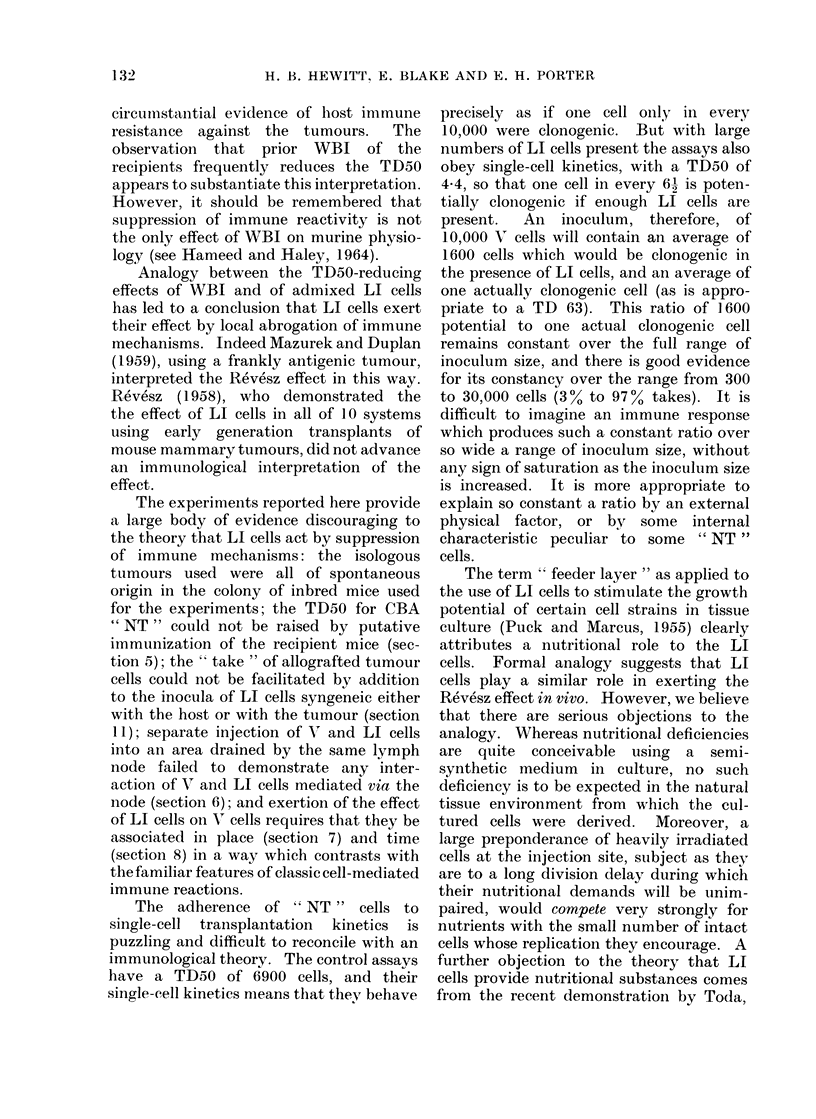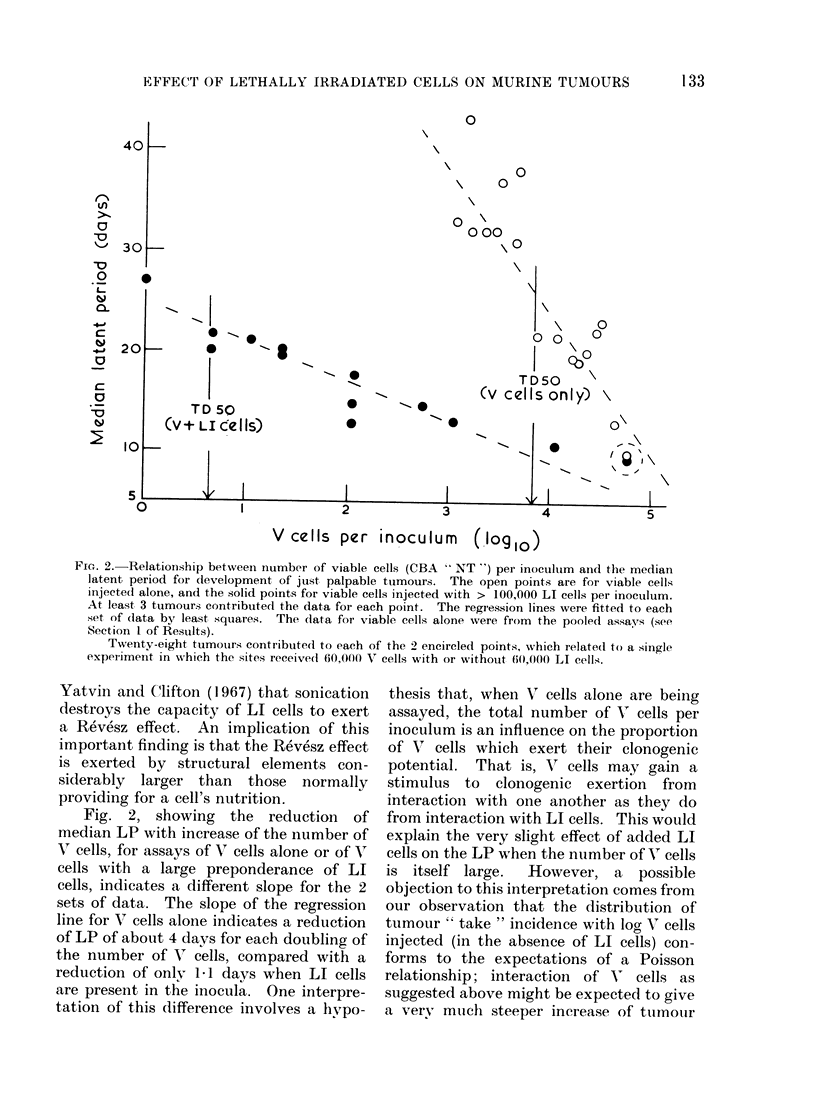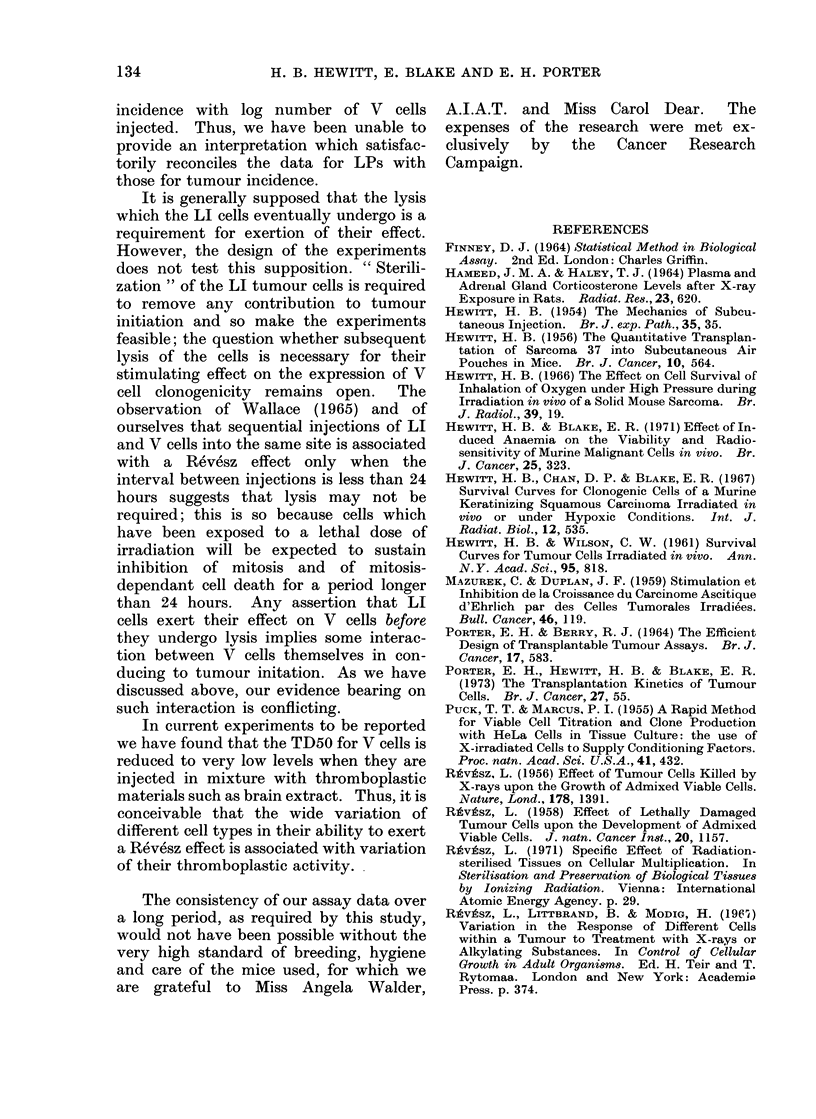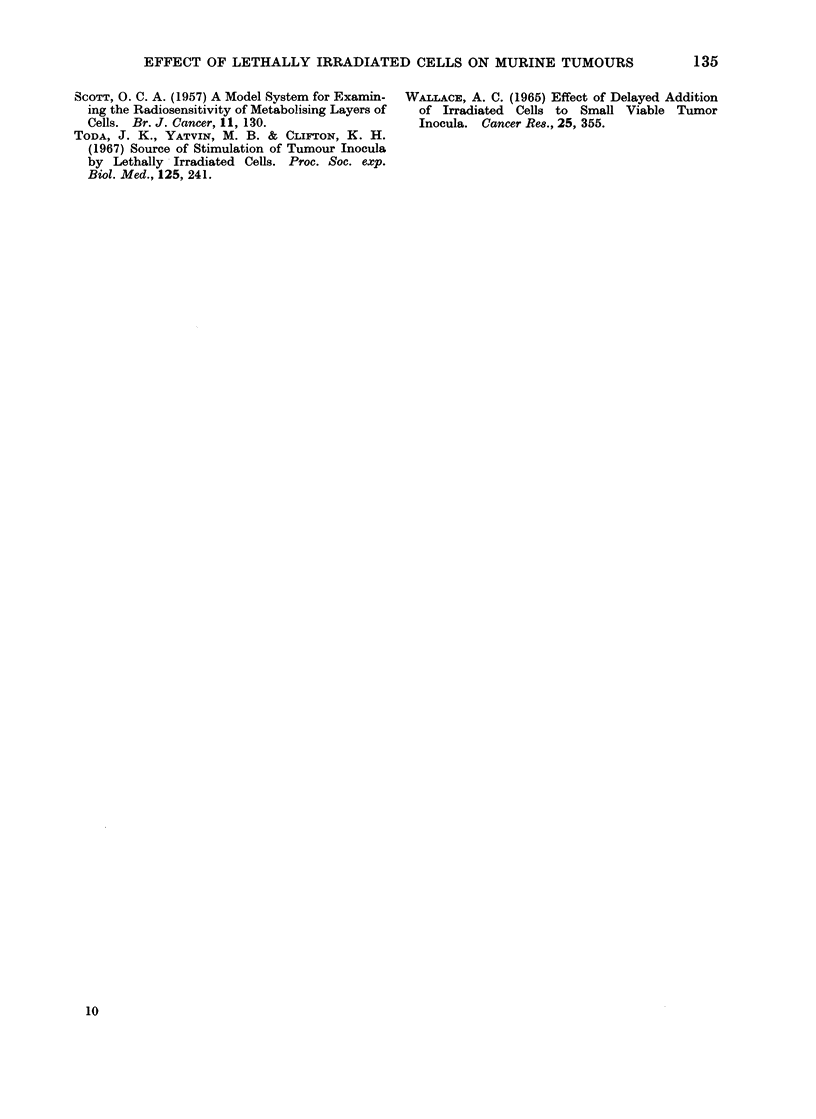# The Effect of Lethally Irradiated Cells on the Transplantability of Murine Tumours

**DOI:** 10.1038/bjc.1973.130

**Published:** 1973-08

**Authors:** H. B. Hewitt, E. Blake, E. H. Porter

## Abstract

Fully quantitative isogeneic transplantation assays of viable (V) cells of a CBA carcinoma showed that the relationship between log inoculum and frequency of tumour “takes” accorded strictly with a Poisson distribution and indicated that 6900 cells were required for 50% takes (TD50). Addition of 10^5^ lethally irradiated (LI) cells of the same tumour to the inocula reduced the TD50 to about 4 cells, yet the Poisson relationship was retained. From this and other data it is concluded that LI cells act by increasing the proportion of viable cells which contribute to tumour initiation; there was no evidence that LI cells affected the rate of proliferation of viable cells. The ability of non-homologous LI cells to reduce the TD50 was widely variable, but LI cells of one allografted tumour were almost as effective as homologous LI cells. Lethally irradiated cells did not assist the “take” of allografted viable tumour cells. Histological comparison revealed no difference of the tissue reaction to inocula of viable and LI cells, and it is questioned whether radiation induced lysis of these latter cells is required for their effect on viable cells. Evidence relating to a hypothesis that viable cells interact with one another as they do with lethally irradiated cells was conflicting.


*Br. J. Cancer* (1973) **28**, 123

### THE EFFECT OF LETHALLY IRRADIATED CELLS ON THE TRANSPLANTABILITY OF MURINE TUMOURS

H. B. HEWITT, E. BLAKE, AND E. H. PORTER

*From the Gray Laboratory, Mount Vernon Hospital, Northwood, Middlesex HA6 2RN, England, and Belvidere Hospital, Glasgow G31 4PG, Scotland*

Received 26 March 1973. Accepted 13 April 1973

**Summary.—Fully quantitative isogeneic transplantation assays of viable (V) cells of a CBA carcinoma showed that the relationship between log inoculum and frequency of tumour " takes " accorded strictly with a Poisson distribution and indicated that 6900 cells were required for 50% takes (TD50). Addition of $10^5$ lethally irradiated (LI) cells of the same tumour to the inocula reduced the TD50 to about 4 cells, yet the Poisson relationship was retained. From this and other data it is concluded that LI cells act by increasing the proportion of viable cells which contribute to tumour initiation; there was no evidence that LI cells affected the rate of proliferation of viable cells. The ability of non-homologous LI cells to reduce the TD50 was widely variable, but LI cells of one allografted tumour were almost as effective as homologous LI cells. Lethally irradiated cells did not assist the " take " of allografted viable tumour cells. Histological comparison revealed no difference of the tissue reaction to inocula of viable and LI cells, and it is questioned whether radiation induced lysis of these latter cells is required for their effect on viable cells. Evidence relating to a hypothesis that viable cells interact with one another as they do with lethally irradiated cells was conflicting.**

IF a sufficient number of viable tumour cells (V cells) are transplanted into an appropriate recipient animal, a tumour will develop. Révész (1956) showed that if a large inoculum of V cells is accompanied by lethally irradiated (LI) cells of the same tumour, the resulting tumour develops earlier and reaches a lethal size earlier than in the absence of LI cells. This effect (the Révész effect) was confirmed by Scott (1957), and extensive subsequent work has been reviewed by Révész (1971).

The Révész effect has been shown to occur with a large number of tumours, both those in which immune reactivity of host against tumour can be demonstrated and those in which it cannot. The effect requires some local association between V cells and LI cells, for if the LI cells are given into the animal's opposite flank no stimulation results. In an irradiated tumour any surviving cells will be intimately mixed with lethally irradiated cells, so that the Révész effect may be important in clinical radiotherapy as well as in the interpretation of survival curves obtained *in vivo* (see Hewitt and Wilson, 1961).

A tumour may appear earlier when LI cells are present, either because more cells have proliferated to form the tumour or because the rate of proliferation of the cells has been increased. Experiments in which a large inoculum size is used cannot discriminate, but Hewitt and Wilson (1961) showed that in at least one tumour LI cells actually increase the number of viable cells which behave in a clonogenic fashion. Table I shows that this occurs in 4 out of 5 syngeneic tumours tested; in the remaining case the number of LI cells may have been insufficient. Table I shows also that there is no constant relationship between the effect of LI cells and the effect of prior whole body irradiation of the recipient animals.

It is clear that the Révész effect is at least partly an effect on the TD50—that is, on the number of V cells that behave in a clonogenic fashion. From Table I, the largest effect on the TD50 is that found with the CBA " NT " tumour, whose TD50 is changed by LI cells by a factor of over 1000. We have therefore used this tumour for the experiments reported in this paper. As each experiment is described, its immediate significance will be briefly mentioned; more general questions will be reserved for the Discussion section.

### MATERIALS AND METHODS

We have found that unrecognized low-grade bacterial infection of a tumour can seriously interfere with its quantitative transplantation. A strictly aseptic technique was followed in all experiments, and all biological materials were kept at 2–5°C except during enzymic treatment of tumour mince.

*Mice and tumours.*—Mice of inbred strains CBA/Ht and WHT/Ht, bred in this laboratory, were used in all experiments; they were aged 2–4 months at the beginning of the experiments. All mice in any one experiment were of the same sex, although we have not found that the sex of the recipients affects the experimental results.

CBA " NT " is a poorly differentiated adenocarcinoma, probably of mammary origin, which arose spontaneously in a female CBA ex-breeder. The tumours used came from serial passages 22–50. Other tumours used are described in footnotes to Tables I and IV.

*Transplantation assays of tumour cells.*—Single-cell suspensions of tumour cells were prepared from enzyme-treated tumour mince as described previously by Hewitt (1966). The density of morphologically intact cells in a suspension was determined by counting in a haemacytometer using phase contrast microscopy, and serial five- or ten-fold dilutions were prepared. Each of a series of selected dilutions was injected subcutaneously in 0·1 ml volumes, usually into 4 sites in 4 mice. Cell counts made after the injections showed no evidence of loss or deterioration of cells during the experiment.

Injected mice were palpated thrice weekly to determine the time of appearance of just palpable tumours; with practice, tumours are detectable when they reach a volume of only a little over 1 $mm^3$. Our " latent periods " (LP) are thus the times from injection to tumours of this size.

*Statistical methods.*—The assays reported here had two purposes: to estimate TD50's and to reveal the relationship between inoculum size and latent period. A wide range of inoculum size was therefore used, though this is not an efficient design for an assay intended solely to estimate a TD50 (see Porter and Berry, 1964). The tumour (CBA "NT") transplants characteristically by single cells, as discussed by Porter, Hewitt and Blake (1973), and the method of Finney (1964) has been used in the analysis of the assays. Finney's method gives a maximum likelihood estimate of the logarithm of the TD50, and an estimate of its standard error. We quote the TD50's themselves, but revert to logarithms (to base 10) when standard errors are required.

Latent periods have not been subjected to any detailed statistical analysis; we present them graphically.

*Preparation of suspensions of LI cells.*—Suspensions were prepared as for viable cells, except that the sedimentation procedure used to produce single-cell suspensions was less stringent and a few clumps of up to 8 cells persisted. The suspensions were exposed in glass vials to 7000–8000 rad of 250 kV x-rays (0·5 mm Cu + 1·0 mm Al filtration) at 300 rad/min. All LI cell suspensions were tested by injection into mice of the strain in which the tumour arose and the mice observed for a year: no tumours have arisen from any LI cell preparations so tested.

*Suspensions of normal tissue cells.*—Normal lymphocytes were obtained by mincing axillary and superficial inguinal lymph nodes. Suspensions of marrow cells were prepared by dispersing the contents of several femora and were irradiated with 250 kV x-rays as described above.

*Technique of whole body irradiation* (WBI).—Groups of 20 mice, placed in a Perspex box, were exposed to $^{60}Co$ gamma rays at a mean distance of 123 cm from the source. The exposure rate, measured with small ionization chambers, was 11 rad/min $\pm$ 4%. A single exposure was given of 500 or 600 rad (the higher dose caused some lethality).

*Abbreviations used.*—V cells: " viable " tumour cells, *i.e.* cells which have the morphology of living cells and which have not been irradiated; LI cells: lethally irradiated cells having the morphology of V cells; TD50: the number of V cells which must be injected to give tumours in 50% of injected sites; WBI: whole body irradiation or whole body irradiated; LP: time between injection of tumour cells and appearance of a just palpable tumour ($\sim$ 1 $mm^3$).

be perfectly valid. But errors in the execution of an experiment will give apparent departures from single-cell transplantation, and it is safer to be suspicious of the experiments in which these anomalous assays served as controls. The remaining experiments can, of course, be confidently referred to the pooled control TD50, whose logarithm (3·84) has a helpfully low standard error (0·038).

TABLE I.—*TD50 Values for Viable (V) Cells of Five Tumours Injected Subcutaneously with or without LI Cells into Normal Mice, or into WBI Mice*

| Tumour | V cells only | V + LI cells (LI cells per inoculum) | V cells in WBI mice |
|---|---|---|---|
| CBA Ca " NT "* | 6900 | 4·4 (125000–151000) | 100 |
| CBA Sa " F "† | 640 | 3·7 (500000) | — |
| WHT Bone Sa I‡ | 190 | 14 (286000) | 13 |
| WHT Bone Sa II‡ | 24000 | 24000 (58000) | 4300 |
| WHT Sq. Ca " D "§ | 21 | 3·7 (2000) | — |

* Described in this paper.
† See Hewitt and Blake (1971).
‡ Anaplastic sarcomata (no longer forming bone).
§ See Hewitt, Chan and Blake (1967).

### RESULTS

### 1. *Subcutaneous TD50's of V cells (controls)*

Subcutaneous assays of V cells alone were performed repeatedly thoughout the period covered by these experiments. Ten of these assays showed single-cell transplantation kinetics, and these assays have been pooled to give the TD50 of 6900 shown in Table I. That they could be pooled shows that the TD50 was stable over this period (it has subsequently dropped to about 2200), and that in the experiments of which they were a part, counting and diluting errors were small compared with the intrinsic statistical errors of the assays themselves.

There were 3 control assays in which the results departed significantly from single-cell transplantation kinetics, though the TD50's were close to 6900. Even with perfect materials it is to be expected that statistical " significance " will be reached by pure chance once in 20 trials, so that any of these assays might in fact

### 2. *Effect on TD50 of LI cells of same tumour*

Fig. I shows the relationship between the number of LI cells added to the inocula of V cells and the TD50 obtained in the relevant assay. In these experiments a constant number of LI cells was added to each inoculum. Seven assays followed single-cell transplantation kinetics, and their TD50's have been plotted as dots with bars representing one standard error on either side. Since the number of LI cells per inoculum was constant in any assay, the ratio LI : V cells varied within each assay; and the detailed results are incompatible with a dependence of the LI effect upon the ratio LI : V cells—this would have given apparent transplantation kinetics very far from single-cell kinetics. Fig. 1 also suggests a threshold somewhere between 5000 LI cells (TD50 near 7000) and 30,000 LI cells (TD50 near 10). There may be another threshold at about 100,000 LI cells, for the 3 assays above this yield a combined TD50 of 4·4 cells

FIG. 1.—Relationship between the TD50 for transplantation assays of CBA "NT" and the number of LI cells added to each inoculum of viable cells.

(the value quoted in Table I); this is just significantly below 10 cells.

Subcutaneously injected cells pack together into a restricted region whose size depends little on the number of cells injected. In this region, V cells will find themselves in an environment of normal connective tissue cells and of LI cells, and clearly the proportion of LI cells in this environment will depend on the number of LI cells in the inoculum rather than on the ratio of LI to V cells in the inoculum. It is therefore tempting to speculate that the effect of LI cells on the TD50 of V cells is exerted only when there are sufficient LI cells to dominate the local environment.

One assay, with 6000 added LI cells, can be interpreted in a way that casts doubt on this speculation. The results were not compatible with single-cell transplantation (*P* less than 0·1%); and the inocula with fewer V cells pointed to lower TD50's, as would be expected universally if the effect depended on the ratio LI : V cells. The separate inocula of this assay pointed to TD50's of 1350, 390 and 81 V cells; these are plotted as crosses in Fig. 1. But an interpretation in terms of the ratio LI : V cells is not necessary: if there is a threshold for the LI effect, then a number of LI cells near the threshold might exert the full effect (TD50 about 10) sometimes, and no effect (TD50 about 7000) at other times, depending on uncontrollable events at the moment of injection. A threshold at 6000 added LI cells does not agree with the results of the assay with 20,000 LI cells:

this showed perfect single-cell transplantation, which is to say that its separate inocula pointed to the same TD50 of 510 V cells. It may be that a threshold exists and its position depends on uncontrolled factors: this would account for the consistent results outside the range 5000–30,000 added LI cells and the inconsistent results within that range.

3. *Histological observations*

The histological changes produced by V and LI cells of CBA "NT" were studied in inoculated sites excised 1, 2, 3, 4, 6, 8 and 10 days after the subcutaneous injection of 300,000 cells. The specimens were fixed in Bouin's solution, sectioned and stained with haematoxylin and eosin. The changes produced by V cells and LI cells were essentially the same: after 1 day a vigorous polymorphonuclear cell reaction can be seen around and between the tumour cells; after 2 and 3 days the inoculum has a necrotic centre, to which the polymorphonuclear cells are confined, and there is a moderate mononuclear cell reaction at the periphery; from 4 to 6 days a moderately dense infiltration of fibroblasts is seen at the periphery and between the tumour cells. At this stage the LI cells are separate from one another, but the V cells have already begun to form small nodules. After 8 days discrete LI cells can still be found, but by 10 days nothing remains at the injection site; meantime the V cells have produced progressively growing tumours with an intense associated fibroblastic reaction.

The early tissue reaction to injected CBA " NT " cells thus does not depend to any striking extent on whether the cells are V or LI cells. This suggests that the effect of LI cells on the V cell TD50 is not exerted by modifying the host's cellular reaction to the inoculum.

4. *Assays in WBI mice*

The TD50 is reduced when the assay is performed in WBI mice. One of the 2 assays which show this effect is suspect, for its control assay was one of the 3 anomalous control assays; but its TD50 (102 cells) agrees excellently with the TD50 (91 cells) of the other assay in WBI mice, which has a perfectly normal control. Both these assays show good agreement with single-cell transplantation kinetics, and it is clear that " NT " cells when assayed in WBI mice have a TD50 of about 100 cells.

If the effect of LI cells were produced by modifying a minor immune reaction of the host against the tumour, it might be expected that the powerful immunosuppressive effect of WBI would leave no effect for LI cells to exert. An assay with 151,000 added LI cells in WBI mice gave a TD50 of 3·3 cells, and one with 141,000 LI cells gave 2·9 cells: these TD50's are not significantly different from each other, or from the 4·4 cells obtained with large numbers of LI cells in unirradiated recipients.

Thus, even in WBI recipient mice, LI cells still have a substantial effect in reducing the TD50 of this tumour; and this suggests that at least a part of the effect of LI cells may not be exerted on an immunological response of the recipients.

5. *Attempt at immunization*

Mice were given 2 intraperitoneal injections of 300,000 LI cells at 7 days' interval. Ten days after the second injection, these mice were used as recipient animals for a subcutaneous assay of V cells. The results were moderately discrepant from single-cell transplantation, as shown by a *chi*-square of 12·8 with 4 d.f., which is beyond the 5% level but not beyond the 1% level. The TD50 in these mice was 3600 cells, to be compared with the pooled control value of 6900 cells, since the control assay of this experiment is a normal member of the pool. Ignoring the moderately high *chi*-square, we find the difference in log TD50's (0·28 log) is 2·3 times its own standard error (0·12 log); and this provides suggestive evidence that the immunization procedure slightly depresses the TD50—*i.e.*, encourages the transplantation of V cells. On the other hand, the raised *chi*-square may hint at a larger but non-uniform effect of immunization; but in either interpretation this technique of immunization certainly does not enable mice to resist tumour cells more strongly.

6. *Nearby subcutaneous LI cells*

In one experiment, 307,000 LI cells were injected, not intimately mixed with the inoculum of V cells, but into a nearby subcutaneous site within the same lymphatic drainage area. Two rather than 4 subcutaneous sites per mouse were used in this assay and in its control. Both assays followed single-cell transplantation kinetics closely. The control assay, however, gave a TD50 of 5250 cells, and this is below the pooled control value of 6900; the significance of the difference is borderline. It seems best, therefore, to regard this cautiously as a self-contained experiment with its own control and not to refer to the pooled controls.

With LI cells nearby the TD50 was 3300 cells, and the difference between this and 5250 cells (0·28 log) is not significant, being barely more than its own standard error (0·19 log). Thus, even a very large number of LI cells exert little or no effect on the TD50 unless they are in actual contact with the V cells. This does not support the idea that LI cells might act by modifying the activities of lymphocytes in the draining lymph nodes. It is of some interest in this context to refer to the results of an experiment in which regional lymph nodes draining implants of this tumour were transplanted whole to CBA mice: 6/20 such nodes gave rise to tumours, suggesting that the regional nodes have no efficient mechanism for destroying cells of this tumour.

7. *Effect of site of injection*

Subcutaneously injected cells are deposited close together near the point of the injecting needle (Hewitt, 1954). If the effect of LI cells requires intimate contact between LI and V cells, it should be reduced or abolished where free dispersion of the inoculum is possible.

Table II shows the results of an assay of V cells made intraperitoneally, with and without LI cells. The assay of V cells alone by this route does not at all follow single-cell transplantation (*chi*-square 63·8 with 4 d.f.; well beyond the 0·1% level). By contrast, when 300,000 LI cells are added the results are perfectly consistent with single-cell transplantation and give a TD50 of 4·5 cells. The standard error of estimate is high (0·24 log), but it is clear that when an excess of LI cells is present the TD50 for intraperitoneal injection of "NT" cells is close to that for subcutaneous injection. The results with intraperitoneal injection of V cells alone are more puzzling.

TABLE II.—*Results of Assay of Viable "NT" Tumour Cells by the Intraperitoneal Route with or without Added LI Cells*

| V cells per inoculum | Tumours/mice injected: V cells only | Tumours/mice injected: V cells with 300000 LI cells |
|---|---|---|
| 45700 | 6/6 | 6/6 |
| 4570 | 2/6 | 6/6 |
| 457 | 2/6 | 6/6 |
| 45·7 | 2/6 | 6/6 |
| 4·57 | 0/6 | 3/6 |

A subcutaneous air pouch (Hewitt, 1956) will allow free dispersal of injected cells, as does the peritoneal cavity, but in other respects will resemble the subcutaneous tissue more than the peritoneum. We have a peliminary experiment with air pouches to report, but not a fully quantitative assay.

Pouches were produced in the dorsal subcutaneous tissue by injecting 3 ml of air through a No. 20 hypodermic needle. The needle track seals itself and the pouch remains in existence for several days. Twenty-two mice received 520 V cells alone into such pouches, and 6 tumours developed with a median latent period of

45 days; there were no pouches with multiple tumours and the same inoculum given subcutaneously without preliminary formation of an air sac produced no tumours in 20 sites. This suggests that the air pouch is a more favourable site for transplantation than the undisturbed subcutaneous tissue.

A further 24 mice with pouches received 520 V cells mixed with 390,000 LI cells. Tumours developed in all 24 pouches with a median latent period of 23 days, and 7 pouches contained multiple tumours. This same inoculum given subcutaneously produced tumours in all of 20 sites. It is clear that LI cells substantially increase the transplantability of V cells in air pouches, though no quantitative statement can be made.

Presumably, cells injected intravenously rapidly lose contact with one another. When 2100 V cells were injected intravenously 0/10 mice developed tumours in the lungs or elsewhere; with 440,000 LI cells added to the inocula, only 1/10 mice developed a tumour. If there is any effect of LI cells on the take of intravenously injected V cells in this system it is evidently small.

The results of these experiments indicate that although some contact between V and LI cells appears to be required for an effect on the TD50, contact does not have to be as intimate as is attained by direct subcutaneous injection of a mixture.

8. *Effect of intraperitoneal injection of V cells and LI cells in sequence*

Table III shows the effect of separation in time between the intraperitoneal injection of 200 V cells and that of 88,000 LI cells. In either sequence a gap of 23 hours is enough to abolish or greatly reduce the effect of LI cells. Wallace (1965), in a similar study using intramuscular implantation of the C3HBA tumour, found that LI cells exerted an effect only if they were given within 24 hours after the injection of V cells; but Révész, Littbrand and Modig (1967) were able to stimulate the growth of spontaneous mouse mammary tumours by injecting LI cells as long as 6 weeks after the injection of V cells.

TABLE III.—*Effect of Sequential Injection of 200 V and 88000 LI Cells by the Intraperitoneal Route*

| Interval (hours) | Tumours/mice injected: V cells first | Tumours/mice injected: HR cells first |
|---|---|---|
| 0 | 10/11 | |
| 3 | 11/12 | 8/11 |
| 23 | 1/12 | 2/12 |
| 47 | * | 1/12 |

* The mice in this group received 400 instead of 200 V cells; the tumour incidence was 3/12.

9. *Effect of different types of cell*

Table IV sets out the effect on the TD50 for viable CBA "NT" cells of adding cells of different types to the inocula. Except for the normal lymphocytes the added cells were lethally irradiated.

The two types of normal tissue cell used (unirradiated lymphocytes and LI bone marrow cells) did not affect the TD50 at all. LI cells from tumours of the WHT line of mouse produced very definite effects; the cells of WHT Ascites Tumour I had a relatively small effect, but those of WHT Ca " MT " had a very considerable effect. It is remarkable that LI cells of these foreign tumours should be effective, as V cells of the same tumours are rejected by CBA hosts; with large numbers of V cells temporary growth may occur but this is always followed by spontaneous regression.

LI cells from CBA Sa " F " reduced the TD50 of viable " NT " cells by a factor of 110 and this is not significantly different from the factor of 170 by which they reduce the TD50 of viable Sa " F " cells (see Table I). The similarity between the two factors is striking but we have insufficient data for interpretation.

LI cells from the CBA Leukaemia " Th " (in ascites form) had a striking effect. The results of the assay were

TABLE IV.—*Effect on the TD50 for CBA " NT " Cells of Adding Unirradiated Lymphocytes or Lethally Irradiated (LI) Cells of Various Types to the Inocula*

| LI cells added | Number per inoculum | TD50 |
|---|---|---|
| ——— Nil (control) | — | 6900 |
| CBA Lymphocytes (unirradiated) | $1 \cdot 6 \times 10^6$ | 7200 |
| CBA Marrow cells | $1 \cdot 3 \times 10^5$ | 6600 |
| WHT Ascites Tumour I† | $6 \times 10^5$ | 1400 |
| WHT Ca " MT "‡ | $7 \cdot 8 \times 10^4$ | 24* |
| CBA Ascites Leukaemia " Th "† | $9 \times 10^5$ | 11–810 (see text) |
| CBA Sa "F "† | $27 \cdot 5 \times 10^4$ | 62 |
| CBA " NT " (same tumour) | $125–151 \times 10^3$ | 4·4 |

* The control TD50 for this assay was only 1800 cells, which is significantly less than the value given by the pooled assays (6900).
† See Hewitt and Blake (1971).
‡ An undifferentiated carcinoma of spontaneous origin.

completely irreconcilable with single-cell transplantation (*chi*-square 4000 with 4 d.f.). The individual groups of the assay pointed to TD50's as far apart as 810 and 11 cells. The inocula with smaller numbers of V cells pointed to lower TD50's, as if it were the ratio of LI "Th" cells to V cells that dominated the effect. But the smallest ratio was 900,000 : 3300 (270 : 1) and this is already a very large ratio. Alternatively the effect of LI " Th " cells might be intrinsically a variable one, or might depend on uncontrolled or even random factors; but in this case it would be a mere coincidence that the lowest TD50 pointed to (11 cells) so strikingly resembles the TD50 obtained in the presence of moderate numbers of LI " NT " cells.

In general, it is clear from Table IV that the normal tissue cells tested have no effect, that LI malignant cells do have an effect, and that LI cells of " NT " itself have the greatest effect on the TD50 of viable " NT " cells. A comparison between lines 5 and 7 of Table IV suggests that LI cells of a foreign (WHT) carcinoma are no less effective than LI cells of a syngeneic (CBA) sarcoma.

10. *Latent periods* (*LP*)

In all the subcutaneous assays the injected sites were palpated thrice weekly. The information given by the assays therefore includes latent periods (from the time of injection to the appearance of tumours of about 1 $mm^3$) for inocula of different numbers of V cells with or without LI cells of the same (CBA " NT ") tumour.

The scatter of LPs is large and tends to increase as the LP increases. Fig. 2 shows the trend of median LP with log number of V cells injected with or without added LI cells ($> 10^5$ per inoculum). Each point is the median LP for at least 3 tumours arising in sites injected with aliquots of the same suspension. At the respective TD50 levels, the median LPs for V cells with and without LI cells are not greatly different (22·5 and 25·5 days respectively). It is also shown that, with V cell numbers approaching $10^5$, the median LP is unaffected by the addition of LI cells. The two encircled points are data for an experiment in which 60,000 V cells were injected with or without an equal number of LI cells; in this experiment each point represents the data for 28 tumours. It is evident that any effect of LI cells on the median latent period for this large number of V cells is small.

The small difference between the median LPs at the widely different TD50 levels for separately pooled assays, considered in relation to our finding that assays for both V cells alone and V cells with LI cells give data conforming to single-cell transplantation kinetics, suggests the following implication: that added LI cells act by increasing the proportion of V cells which exert their clonogenicity rather than by increasing the rate of proliferation of the V cells.

The lines drawn through the 2 sets of data points have been fitted by the least squares method; interpretation of their difference of slope will be attempted in the Discussion.

11. *Lethally irradiated cells and allografted viable tumour cells*

When the cells of a CBA tumour are injected into WHT mice (or WHT tumour into CBA mice) a typical immune reaction of host against tumour is seen; a sufficient number of injected cells may give a temporary tumour but spontaneous regression always occurs. If LI cells could abolish or diminish this familiar rejection response, then it would be reasonable to assume that their effect on the transplantability of isografted tumours might be exerted on some minor immune reaction.

In one experiment, V cells of a CBA tumour were assayed subcutaneously in WHT mice with or without 400,000 LI cells of the same (CBA) tumour. All the resulting tumours regressed before the nineteenth day. With V cells alone, the tumours produced by larger inocula appeared earlier and regressed earlier than those produced by smaller inocula. There were fewer tumours when the V cells were accompanied by LI cells, and regression was earlier. The TD50's for temporary takes were 11,000 for V cells alone and 35,000 for V cells with LI cells. Thus, in this situation LI cells provide a stimulus for immune rejection, and are very far from interfering with it.

In another experiment, V cells of a WHT tumour were injected into CBA mice with and without LI cells of a CBA tumour. The LI cells affected neither the number of temporary tumours nor their time of regression. This is in sharp contrast to the large effect of foreign LI cells on the TD50 when the tumour is of the same strain as the host.

Thus, we have found no evidence that addition of isogeneic or foreign LI cells to the inocula assists the transplantability of allografted viable tumour cells.

12. *Failures to take with large inocula*

We have encountered 3 examples from assay data of a single failure to " take " among a group of 16 sites which received an inoculum between 30 and 300 times larger than the TD50 obtained in the assay. The risk of such an occurrence arising by chance is very low indeed (less than $10^{-7}$) and we cannot accept these 3 instances as random. Unexpected failures to " take " could, of course, arise from a technical error but we believe that they may represent a real biological effect.

With large inocula, the LP is relatively short but the individual values within a uniform group of injected sites are fairly widely scattered; this is not well shown in Fig. 2 because the points plotted represent median, not individual, values. It is commonly found that mice which have received large inocula and which subsequently require sacrifice for humane reasons, have 3 very large tumours and one very small one; at this stage the tumour bearing mice may exhibit signs of constitutional depletion, as indicated, for example, by anaemia. The smallest tumour may be only just palpable at the time of sacrifice but histological study of such delayed tumours has confirmed that the mass is viable tumour. It is clear that appearance of a tumour that is delayed in its early growth may be repressed entirely if constitutional depletion of the host ensues before it reaches a palpable size. It is significant that we do not observe such small tumours in mice which receive intermediate sized inocula and which do not sustain such early constitutional depletion.

### DISCUSSION

High TD50 values for the transplantation of tumours are commonly cited as circumstantial evidence of host immune resistance against the tumours. The observation that prior WBI of the recipients frequently reduces the TD50 appears to substantiate this interpretation. However, it should be remembered that suppression of immune reactivity is not the only effect of WBI on murine physiology (see Hameed and Haley, 1964).

Analogy between the TD50-reducing effects of WBI and of admixed LI cells has led to a conclusion that LI cells exert their effect by local abrogation of immune mechanisms. Indeed Mazurek and Duplan (1959), using a frankly antigenic tumour, interpreted the Révész effect in this way. Révész (1958), who demonstrated the the effect of LI cells in all of 10 systems using early generation transplants of mouse mammary tumours, did not advance an immunological interpretation of the effect.

The experiments reported here provide a large body of evidence discouraging to the theory that LI cells act by suppression of immune mechanisms: the isologous tumours used were all of spontaneous origin in the colony of inbred mice used for the experiments; the TD50 for CBA "NT" could not be raised by putative immunization of the recipient mice (section 5); the "take" of allografted tumour cells could not be facilitated by addition to the inocula of LI cells syngeneic either with the host or with the tumour (section 11); separate injection of V and LI cells into an area drained by the same lymph node failed to demonstrate any interaction of V and LI cells mediated *via* the node (section 6); and exertion of the effect of LI cells on V cells requires that they be associated in place (section 7) and time (section 8) in a way which contrasts with the familiar features of classic cell-mediated immune reactions.

The adherence of "NT" cells to single-cell transplantation kinetics is puzzling and difficult to reconcile with an immunological theory. The control assays have a TD50 of 6900 cells, and their single-cell kinetics means that they behave precisely as if one cell only in every 10,000 were clonogenic. But with large numbers of LI cells present the assays also obey single-cell kinetics, with a TD50 of 4·4, so that one cell in every $6\frac{1}{2}$ is potentially clonogenic if enough LI cells are present. An inoculum, therefore, of 10,000 V cells will contain an average of 1600 cells which would be clonogenic in the presence of LI cells, and an average of one actually clonogenic cell (as is appropriate to a TD 63). This ratio of 1600 potential to one actual clonogenic cell remains constant over the full range of inoculum size, and there is good evidence for its constancy over the range from 300 to 30,000 cells (3% to 97% takes). It is difficult to imagine an immune response which produces such a constant ratio over so wide a range of inoculum size, without any sign of saturation as the inoculum size is increased. It is more appropriate to explain so constant a ratio by an external physical factor, or by some internal characteristic peculiar to some "NT" cells.

The term "feeder layer" as applied to the use of LI cells to stimulate the growth potential of certain cell strains in tissue culture (Puck and Marcus, 1955) clearly attributes a nutritional role to the LI cells. Formal analogy suggests that LI cells play a similar role in exerting the Révész effect *in vivo*. However, we believe that there are serious objections to the analogy. Whereas nutritional deficiencies are quite conceivable using a semi-synthetic medium in culture, no such deficiency is to be expected in the natural tissue environment from which the cultured cells were derived. Moreover, a large preponderance of heavily irradiated cells at the injection site, subject as they are to a long division delay during which their nutritional demands will be unimpaired, would *compete* very strongly for nutrients with the small number of intact cells whose replication they encourage. A further objection to the theory that LI cells provide nutritional substances comes from the recent demonstration by Toda,

FIG. 2.—Relationship between number of viable cells (CBA " NT ") per inoculum and the median latent period for development of just palpable tumours. The open points are for viable cells injected alone, and the solid points for viable cells injected with > 100,000 LI cells per inoculum. At least 3 tumours contributed the data for each point. The regression lines were fitted to each set of data by least squares. The data for viable cells alone were from the pooled assays (see Section 1 of Results).

Twenty-eight tumours contributed to each of the 2 encircled points, which related to a single experiment in which the sites received 60,000 V cells with or without 60,000 LI cells.

Yatvin and Clifton (1967) that sonication destroys the capacity of LI cells to exert a Révész effect. An implication of this important finding is that the Révész effect is exerted by structural elements considerably larger than those normally providing for a cell's nutrition.

Fig. 2, showing the reduction of median LP with increase of the number of V cells, for assays of V cells alone or of V cells with a large preponderance of LI cells, indicates a different slope for the 2 sets of data. The slope of the regression line for V cells alone indicates a reduction of LP of about 4 days for each doubling of the number of V cells, compared with a reduction of only 1·1 days when LI cells are present in the inocula. One interpretation of this difference involves a hypothesis that, when V cells alone are being assayed, the total number of V cells per inoculum is an influence on the proportion of V cells which exert their clonogenic potential. That is, V cells may gain a stimulus to clonogenic exertion from interaction with one another as they do from interaction with LI cells. This would explain the very slight effect of added LI cells on the LP when the number of V cells is itself large. However, a possible objection to this interpretation comes from our observation that the distribution of tumour " take " incidence with log V cells injected (in the absence of LI cells) conforms to the expectations of a Poisson relationship; interaction of V cells as suggested above might be expected to give a very much steeper increase of tumour incidence with log number of V cells injected. Thus, we have been unable to provide an interpretation which satisfactorily reconciles the data for LPs with those for tumour incidence.

It is generally supposed that the lysis which the LI cells eventually undergo is a requirement for exertion of their effect. However, the design of the experiments does not test this supposition. " Sterilization " of the LI tumour cells is required to remove any contribution to tumour initiation and so make the experiments feasible; the question whether subsequent lysis of the cells is necessary for their stimulating effect on the expression of V cell clonogenicity remains open. The observation of Wallace (1965) and of ourselves that sequential injections of LI and V cells into the same site is associated with a Révész effect only when the interval between injections is less than 24 hours suggests that lysis may not be required; this is so because cells which have been exposed to a lethal dose of irradiation will be expected to sustain inhibition of mitosis and of mitosis-dependant cell death for a period longer than 24 hours. Any assertion that LI cells exert their effect on V cells *before* they undergo lysis implies some interaction between V cells themselves in conducing to tumour initation. As we have discussed above, our evidence bearing on such interaction is conflicting.

In current experiments to be reported we have found that the TD50 for V cells is reduced to very low levels when they are injected in mixture with thromboplastic materials such as brain extract. Thus, it is conceivable that the wide variation of different cell types in their ability to exert a Révész effect is associated with variation of their thromboplastic activity.

The consistency of our assay data over a long period, as required by this study, would not have been possible without the very high standard of breeding, hygiene and care of the mice used, for which we are grateful to Miss Angela Walder, A.I.A.T. and Miss Carol Dear. The expenses of the research were met exclusively by the Cancer Research Campaign.

### REFERENCES

FINNEY, D. J. (1964) *Statistical Method in Biological Assay.* 2nd Ed. London: Charles Griffin.

HAMEED, J. M. A. & HALEY, T. J. (1964) Plasma and Adrenal Gland Corticosterone Levels after X-ray Exposure in Rats. *Radiat. Res.*, **23**, 620.

HEWITT, H. B. (1954) The Mechanics of Subcutaneous Injection. *Br. J. exp. Path.*, **35**, 35.

HEWITT, H. B. (1956) The Quantitative Transplantation of Sarcoma 37 into Subcutaneous Air Pouches in Mice. *Br. J. Cancer*, **10**, 564.

HEWITT, H. B. (1966) The Effect on Cell Survival of Inhalation of Oxygen under High Pressure during Irradiation *in vivo* of a Solid Mouse Sarcoma. *Br. J. Radiol.*, **39**, 19.

HEWITT, H. B. & BLAKE, E. R. (1971) Effect of Induced Anaemia on the Viability and Radiosensitivity of Murine Malignant Cells *in vivo*. *Br. J. Cancer*, **25**, 323.

HEWITT, H. B., CHAN, D. P. & BLAKE, E. R. (1967) Survival Curves for Clonogenic Cells of a Murine Keratinizing Squamous Carcinoma Irradiated *in vivo* or under Hypoxic Conditions. *Int. J. Radiat. Biol.*, **12**, 535.

HEWITT, H. B. & WILSON, C. W. (1961) Survival Curves for Tumour Cells Irradiated *in vivo*. *Ann. N.Y. Acad. Sci.*, **95**, 818.

MAZUREK, C. & DUPLAN, J. F. (1959) Stimulation et Inhibition de la Croissance du Carcinome Ascitique d'Ehrlich par des Celles Tumorales Irradiées. *Bull. Cancer*, **46**, 119.

PORTER, E. H. & BERRY, R. J. (1964) The Efficient Design of Transplantable Tumour Assays. *Br. J. Cancer*, **17**, 583.

PORTER, E. H., HEWITT, H. B. & BLAKE, E. R. (1973) The Transplantation Kinetics of Tumour Cells. *Br. J. Cancer*, **27**, 55.

PUCK, T. T. & MARCUS, P. I. (1955) A Rapid Method for Viable Cell Titration and Clone Production with HeLa Cells in Tissue Culture: the use of X-irradiated Cells to Supply Conditioning Factors. *Proc. natn. Acad. Sci. U.S.A.*, **41**, 432.

RÉVÉSZ, L. (1956) Effect of Tumour Cells Killed by X-rays upon the Growth of Admixed Viable Cells. *Nature, Lond.*, **178**, 1391.

RÉVÉSZ, L. (1958) Effect of Lethally Damaged Tumour Cells upon the Development of Admixed Viable Cells. *J. natn. Cancer Inst.*, **20**, 1157.

RÉVÉSZ, L. (1971) Specific Effect of Radiation-sterilised Tissues on Cellular Multiplication. In *Sterilisation and Preservation of Biological Tissues by Ionizing Radiation.* Vienna: International Atomic Energy Agency. p. 29.

RÉVÉSZ, L., LITTBRAND, B. & MODIG, H. (1967) Variation in the Response of Different Cells within a Tumour to Treatment with X-rays or Alkylating Substances. In *Control of Cellular Growth in Adult Organisms.* Ed. H. Teir and T. Rytomaa. London and New York: Academic Press. p. 374.

SCOTT, O. C. A. (1957) A Model System for Examining the Radiosensitivity of Metabolising Layers of Cells. *Br. J. Cancer*, **11**, 130.

TODA, J. K., YATVIN, M. B. & CLIFTON, K. H. (1967) Source of Stimulation of Tumour Inocula by Lethally Irradiated Cells. *Proc. Soc. exp. Biol. Med.*, **125**, 241.

WALLACE, A. C. (1965) Effect of Delayed Addition of Irradiated Cells to Small Viable Tumor Inocula. *Cancer Res.*, **25**, 355.